# Terc is dispensable for most of the short-term HPV16 oncogene-mediated phenotypes in mice

**DOI:** 10.1371/journal.pone.0196604

**Published:** 2018-04-26

**Authors:** Charis Achilleos, Stella Michael, Katerina Strati

**Affiliations:** Department of Biological Sciences, University of Cyprus, Nicosia, Cyprus; International Centre for Genetic Engineering and Biotechnology, ITALY

## Abstract

High-risk human papillomaviruses (HPVs) have been shown *in vitro* to impinge on telomere homeostasis in a number of ways. However, the *in vivo* interaction of viruses with the telomere homeostasis apparatus has not been previously explored. Since E6 and E7 are the main viral oncogenes and key for viral replication, we have explored here the short-term phenotypes of the genes in the context of defective telomere homeostasis. We examined the short-term phenotypes of E6 and E7 in a context where the Terc component of the telomerase holoenzyme was knocked out. We determined that Terc was dispensable for most oncogene-mediated phenotypes. Surprisingly, E7-mediated reduction of label retaining cells was found to be in part dependent on the presence of Terc. Under the conditions examined here, there appears to be no compelling evidence Terc is required for most short-term viral oncogene mediated phenotypes. Further studies will elucidate its role in longer-term phenotypes.

## Introduction

Human papillomaviruses are considered to be important human pathogens and model systems of viral carcinogenesis. High risk HPVs have been associated with the development of the majority of cervical cancers, a subset of head and neck cancers, as well as other anogenital cancers. HPV-associated cancers are thought to account for >5% of the worldwide cancer burden [1].

In HPV-associated malignancies carcinogenesis is driven, in large part, by the continuous expression of the viral oncogenes E6 and E7 [2, 3]. These oncogenes have no known enzymatic actions but mediate their profound effects in infected cells by engaging important cellular proteins including the tumor suppressors p53 and pRb [4–7]. Interactions with cellular targets are critical determinants of viral lifecycle and are sometimes important in viral driven carcinogenesis, which is linked to persistent infection and often viral integration. There is credible evidence which suggests that telomerase reactivation and changes in telomere homeostasis are a contributing factor to the oncogenic effects of HPV.

Telomerase reactivation is observed in the overwhelming majority of cancers [8] and has been demonstrated to be the key mechanism, which counteracts cellular senescence and apoptosis, brought on by telomere erosion in highly proliferative malignancies. In normal cells telomerase activity is high in embryonic and tissue stem cells but is gradually diminished during the normal differentiation process to low or undetectable levels [9, 10]. Reactivation during the oncogenic process, often during late stages, has been described to occur by means of transcriptional and/or translational upregulation of the enzymatic component of the telomerase holoenzyme Tert [11]. In a minority of cancers (10–15%) [12] telomeres are elongated via telomerase-independent Alternative Lengthening of Telomeres (ALT).

Intriguingly high-risk HPV oncogenes have been shown by *in vitro* studies to modulate telomere homeostasis in a variety of ways. Both E6 and E7 have been implicated in regulating telomere length by means of telomerase activation and ALT respectively. The HPV16 E6 oncoprotein affects telomerase activity by increasing the transcriptional levels of the human telomerase reverse transcriptase (hTERT) component of telomerase, an essential step for cell immortalization [13]. This can be achieved either by inducing the activation of the Tert promoter, via its interactions with the promoter’s activator proteins (eg. Myc and NFX1-123) or repressor proteins (eg. NFX1-91), or by directly binding to the Tert protein [13–17] and telomeric repeats. In HeLa cells re-expression of either E6 or E7, after their removal, leads to increased hTERT [18]. Studies from Kiyono et al. showed that the E6-induced activation of telomerase activity requires the combined effect of the E7-induced inactivation of the Rb pathway for immortalization of both fibroblasts and keratinocytes in culture [19]. Other studies demonstrated that E7 can increase hTERT promoter-driven expression and augment telomerase activity driven by HPV E6 [20] and can immortalize keratinocytes by cooperating with Tert even when defective for telomere maintenance [21].

However, the multitude of ways in which the HPV oncogenes have been shown to modulate telomere homeostasis point to another intriguing possibility: that this is a phenomenon reflective of evolutionary adaptation of the virus [13]. This of course would suggest that telomere homeostasis is important not only during later stages of carcinogenesis but also during the viral lifecycle (and potentially contributing to earlier stages of carcinogenesis). For example such an alteration in telomere homeostasis, driving cells towards a more stem-like phenotype, could allow viral persistency a critical initial step for carcinogenesis.

This study addresses the role of telomere homeostasis on the effects of the HPV16 oncogenes on the stratified epithelia of the skin, as previously reported [22], using transgenic mice expressing E6 and E7 under the K14 promoter [23] as well as mice deficient for the expression of the telomerase RNA component [24]. We examined the *in vivo* interplay of the HPV16 E6 and E7 oncogenes with the telomerase complex and its effects on the short-term phenotypes caused by the viral oncogenes on target tissues. These phenotypes correlate both to events important to early carcinogenesis as well as to the viral lifecycle and shed light on the importance of the telomerase complex during viral replication and disease.

## Materials and methods

### Ethics statement

This study was carried out in strict accordance with the recommendations in the Guidelines for the Protection of Laboratory Animals of the Republic of Cyprus. The animal facility is licensed by the Veterinary Services (Republic of Cyprus Ministry of Agriculture and Natural Resources), the government body in charge of approving and overseeing laboratory animal work in Cyprus (license number CY.EXP.105) and the protocol was approved by the same authority (License number CY/EXP/PR.L1/2013). The work described here does not involve procedures requiring anesthesia. Mice were sacrificed at the specified ages. We adhere to acceptable euthanasia guidelines using a CO_2_ chamber.

### Mice

The mouse strains used in the experiments were obtained from the crosses of K14E6/E7TTL referred to as K14E6, K14E7/E6TTL referred to K14E7 as previously described [23, 25] and Terc+/- mice [24]. The K14E6 and K14E7 mice were of a pure FVB/N genetic background and Terc+/- mice were on a pure C57BL/6 genetic background. All the genotypes were confirmed by means of PCR.

### Generation of mice expressing the HPV16 oncogenes in the presence or absence of Terc

For assessing the role of the absence of Terc and thus of telomerase on the HPV-expressing epithelia, mice expressing the HPV16 oncogenes in the presence or absence of Terc were generated. Heterozygous Terc+/- mice, were crossed with K14E6 or K14E7 mice to generate the F1 K14E6Terc+/- and K14E7Terc+/-. These F1 mice were subsequently crossed with Terc+/- to generate the first generation (G1) of mice deficient for the production of Terc. All the genotypes obtained from the crosses are listed on Table 1. The crossing scheme was consistent for all genotypes obtained. All mice genotypes were confirmed by means of PCR, using DNA extracted from tail clippings.

**Table 1:** Experimental mice used.

Genotype	Transgene
NTG Terc+/+	None
K14E7 Terc+/+	E7
K14E6 Terc+/+	E6
K14NTG Terc-/-	None
K14E7 Terc-/-	E7
K14E6 Terc-/-	E6

The crossing scheme generated mice expressing telomerase (Terc+/+) and mice deficient for telomerase (Terc-/-). These mice were either non-transgenic for the viral oncogenes (NTG) or transgenic for the oncogenes (K14E6 and K14E7).

### BrdU incorporation

5-Bromo-2-deoxyuridine (BrdU) was administered peritoneally in mice at a final concentration of 50 mg/kg as first described previously [26]. For pulse chase experiments ten-day-old mice received an injection every 12 h for a total of four doses. They were euthanized 60 days after injections.

### Immunohistochemistry

Mice were sacrificed, and tissues obtained were fixed in 4% paraformaldehyde overnight at 4°C. Dehydration of the samples was performed in a graded series of ethanol concentrations and xylene before they were embedded in paraffin wax. Sections of 10 μm thickness were obtained using a microtome and left overnight to dry at room temperature. Samples were deparaffinised in xylene and rehydrated in a graded series of ethanol solutions. Antigen retrieval was done in a microwave using 10 mM citrate buffer and for BrdU immunohistochemistry, samples were also incubated for 20 min in 2 M HCl. Blocking and antibody incubations were variable and optimal for each different antibody used. Primary antibodies used include: 1:100 BrdU (Abcam, ab6326, rat monoclonal), 1:500 K15 (SantaCruz, sc-47697, mouse monoclonal), 1:100 PCNA (SantaCruz, sc-25280, mouse monoclonal). Following primary antibody incubation samples were washed in PBS. The following secondary antibodies were used: Cy3-streptavidin, biotin-rat and biotin -mouse all from Jackson ImmunoResearch and also Vectastain universal secondary (Vector laboratories, #016-160-084). All images were acquired using a Zeiss Axio Observer.A1 microscope. Quantification was performed in a blinded fashion.

### Fluorescent in situ hybridization (FISH) assay

Samples were deparaffinised in xylene and rehydrated in a graded series of ethanol solutions. They were then treated with HCl/Pepsin for 10 min at 37°C, washed in PBS and then fixed in 4% paraformaldehyde for 2 min, washed in PBS and dehydrated in a graded series of ethanol concentrations. The slides were air-dried and the DNA was denaturated for 2 min at 80°C and then hybridized for 2 h with Cy3-conjugated peptide nucleic acid (PNA) probe in the dark at room temperature for 2 h. Samples were washed in wash solution (Final concentration: 70% formamide, 10mM Tris, 0.1% BSA) twice for 15 min and three times for 5 min in TBS-Tween 0.08%. After the washes, the slides were dehydrated in a graded series of ethanol solutions and mounted with DAPI. Samples were analyzed on a Zeiss LSM 710 Axiovert confocal microscope using a 63× Plan-Neofluar 1.4 NA oil immersion objective lens. Images were analyzed with Axiovision 4.2 software and processed using TFL-Telo V2.

### Telomeric repeat amplification protocol (TRAP) assay

Trap assay was performed using the TRAPeze® Telomerase Detection Kit (S7700-KIT; Millipore). Briefly, fresh skin tissue was obtained and floated with the dermis side down in 0.25% trypsin at 4°C O/N. On the next day, the dermis was peeled off and the epidermis was homogenized while kept on ice. CHAPS lysis buffer was added (200μl per 10μg of tissue) and samples were incubated on ice for 30 min. Then, 160μL of the supernatant was collected, and the protein concentration was determined. A reaction mix containing TRAP buffer (20 mM Tris–HCl, pH 8.3, 1.5 mM MgCl_2_, 63 mM KCl, 0.05% (v/v) Tween-20, 1 mM EDTA, and 0.01% BSA; TRAPeze telomerase detection kit), supplemented with dNTP mix, TS primer, TRAP primer mix, dH_2_O, Taq polymerase at indicated concentrations was prepared and incubated with the samples at 30°C for 30 min. PCR was used for amplication (94°C for 30 s, 59°C for 30 s, 72°C for 1 min for 30 cycles and 72°C for 7 min in a thermocycler. PCR samples were run on a 10% (w/v) native-PAGE gel in 0.5XTBE for 1 h at 150 V. After electrophoresis, the gel was stained with ethidium bromide for 10 min at room temperature. The telomerase activity was estimated by measuring the intensity of the bands using ImageJ software.

### Statistical tests

To determine the statistical significance between the genotypes in each experiment, 3 mice of each genotype were used and 60–80 hair follicles were counted. Statistical analysis was done using “Mstat” software (version 5.5.3, McArdle Laboratory for Cancer Research, University of Wisconsin–Madison [http://mcardle.oncology.wisc.edu/mstat/]). Results were compared using a Wilcoxon rank sum test. For all statistical tests differences were considered statistically significant at p≤0.05.

## Results

### Expression of the HPV16 E6 oncogene leads to increased telomerase activity *in vivo*

**Fig 1:**
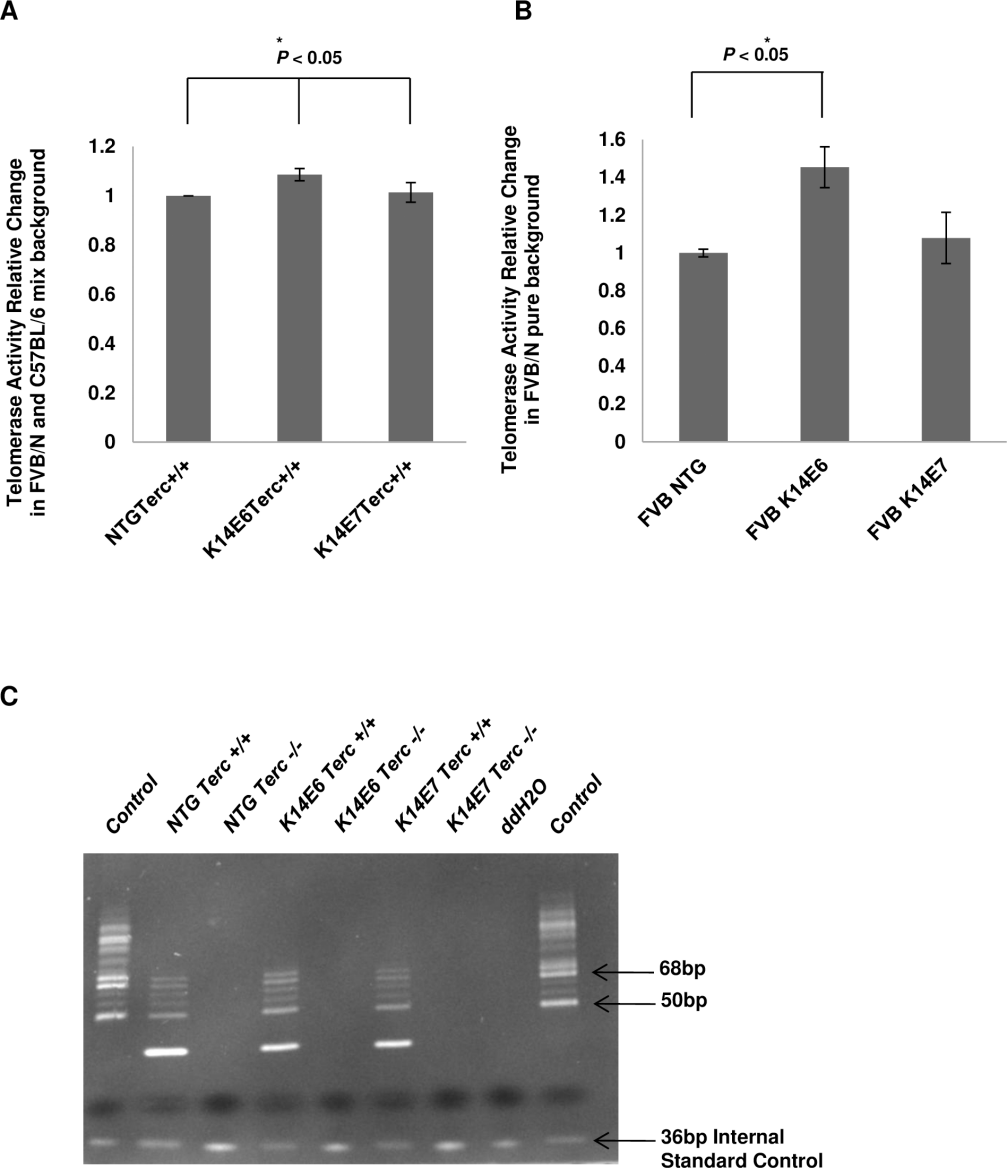
Expression of the HPV16 E6 oncogene increases telomerase activity *in vivo*. (A) and (B) The telomerase activity was determined using ImageJ software analysis and plotted for each genotype (columns); bars, SD. The experiments were done using three mice from each genotype and all statistical comparisons were performed using a two-sided Wilcoxon rank sum test. Statistical significance was observed between E6 and NTG or E7 mice. (NTG; Non Transgenic) (C) Representative native PAGE gel image of the telomerase products extracted from skin tissue and amplified using the Trap assay.

To assess the levels of telomerase activity in mouse epithelia expressing the HPV16 oncogenes we performed TRAP assays. Using epithelial extracts from K14E6 and K14E7 mice we found that a low but statistically significant increase in telomerase activity was seen in epithelia for K14E6 animals, a result consistent in both mixed (Fig 1A) and pure (Fig 1B) genetic backgrounds. This is the first time that such an increase is shown *in vivo* and this result is consistent with previously reported telomerase upregulation in human keratinocytes transduced with HPV16 oncogenes [27]. Thus, we sought to determine potential roles of telomerase in short-term phenotypes of transgenic animals.

To study the canonical-roles of telomerase we crossed animals transgenic for the HPV16 oncogenes with animals in which the RNA component of the telomerase holoenzyme (Terc) has been knocked-out. It has been previously shown that Terc knockout mice lack detectable telomerase activity [24]. To confirm the telomerase status of the mice genotypes used in our experiments, telomere repeat amplification protocol (TRAP) was used. Mice lacking the Terc component of telomerase showed no telomerase activity as expected (Fig 1C).

### Telomeric length is unaffected by HPV16 oncogene expression *in vivo*

**Fig 2:**
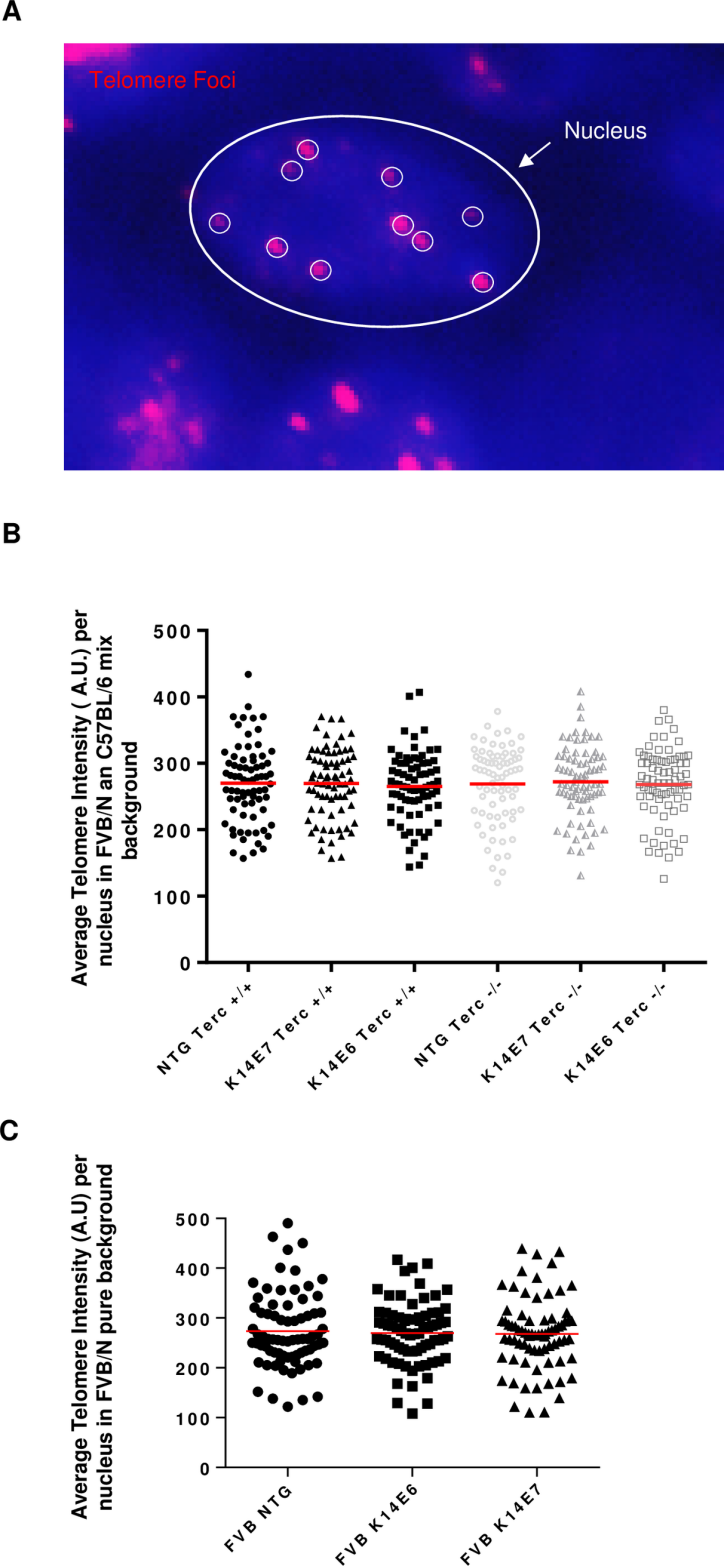
Expression of the HPV16 oncogenes does not affect telomeric length irrespective of the presence or absence of Terc. (A) Representative immunofluorescent image of telomeres (depicted by small white circles) using fluorescent in situ hybridisation assay (FISH) on the tail of 71 day-old mice. The nucleus is shown by an arrow. (B) and (C) The average length of the telomeres was determined by measuring the intensity of the telomeric foci using TFL-telo software in FVB/N and C57BL/6 mixed background (B) and FVB/N pure background (C). ~75 nuclei were counted for each genotype and the mean average was plotted for each genotype (columns); bars, SD. A.U; Arbitrary Units The experiments were done using three mice from each genotype and all statistical comparisons were performed using a two-sided Wilcoxon rank sum test.

To examine the effect of the HPV16 E6 and E7 oncogene expression on telomere length, tissue from mice with defective and wild type Terc were subjected to Q-FISH (Fig 2A) and the average telomere length of telomeres from 75 nuclei was compared among all genotypes (Fig 2B). Results revealed no statistical differences among the different samples. Results were verified using mice on a pure FVB/N background (Fig 2C).

The telomerase holoenzyme is responsible for telomere elongation and under normal conditions preferentially acts on critically short telomeres slowing down their transition into senescence. It is frequently found to be upregulated in cancers, presumably to maintain a sufficiently long telomeric length despite the constant rounds of cell division common in malignancy. Thus, the activation of telomerase in tumors is thought to be a late event in carcinogenesis. These findings could explain why the absence of Terc and thus of telomerase in mice of sufficiently long telomeres did not affect the telomere length in any of the genotypes tested.

### Canonical roles of telomerase are dispensable for HPV16 oncogene-mediated increase in epithelial proliferation

**Fig 3:**
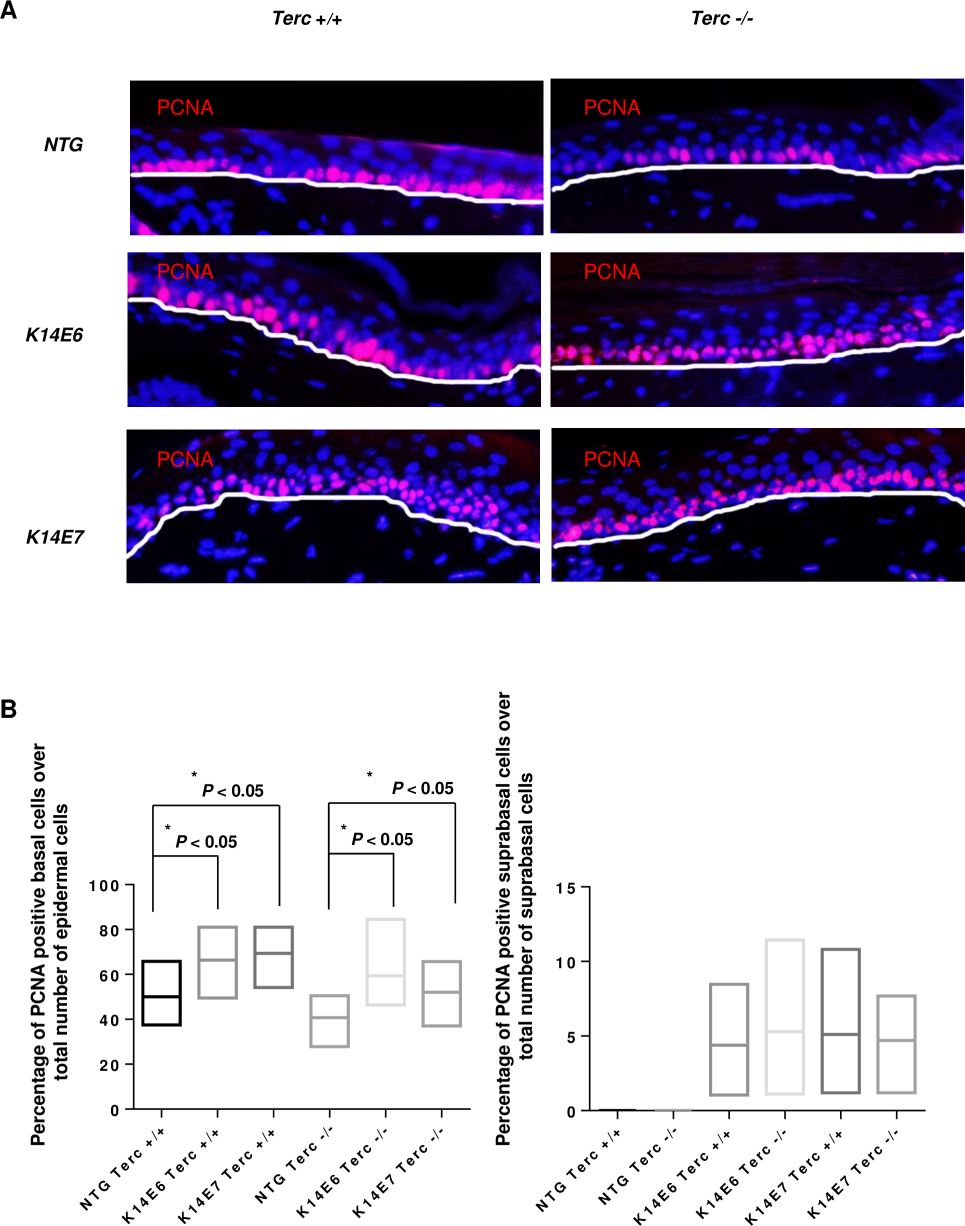
Expression of the HPV16 oncogenes increases the proliferative ability of the epithelium irrespective of the presence or absence of Terc. (A) Representative immunofluorescent images of the tail epithelium showing PCNA positive cells (red). Counterstaining was done with DAPI (blue). The white line indicates the basal membrane in each image. (B) The proliferative ability was determined by counting ~25 different regions of the basal (left) and suprabasal (right) layers. Mice were 71 days old. The positive cells were counted for each genotype and the percentage of positive cells over the total number of cells was plotted (columns); bars, SD. The experiments were done using three mice from each genotype and all statistical comparisons were performed using a two-sided Wilcoxon rank sum test. As expected, no positive PCNA cells were detected in the suprabasal layers of non-transgenic animals.

It has been previously shown that the HPV16 E6 and E7 oncogenes cause an increase in proliferation as marked by increased PCNA detection in anagen hair follicles [22]. Our results have verified previous findings, as an increase in PCNA positive cells in the mouse tail epithelium (Fig 3A), is seen in both basal (Fig 3B) and suprabasal (Fig 3C) layers. However, Terc was dispensable for these phenotypes suggesting that the HPV-driven proliferation of the epithelium is not connected to the telomerase status of the epithelium. Whether the Tert component of the telomerase complex is involved in the HPV-driven proliferation is still unknown.

### Terc is dispensable for E6 but not E7-mediated effects on hair follicle label-retaining cells

**Fig 4:**
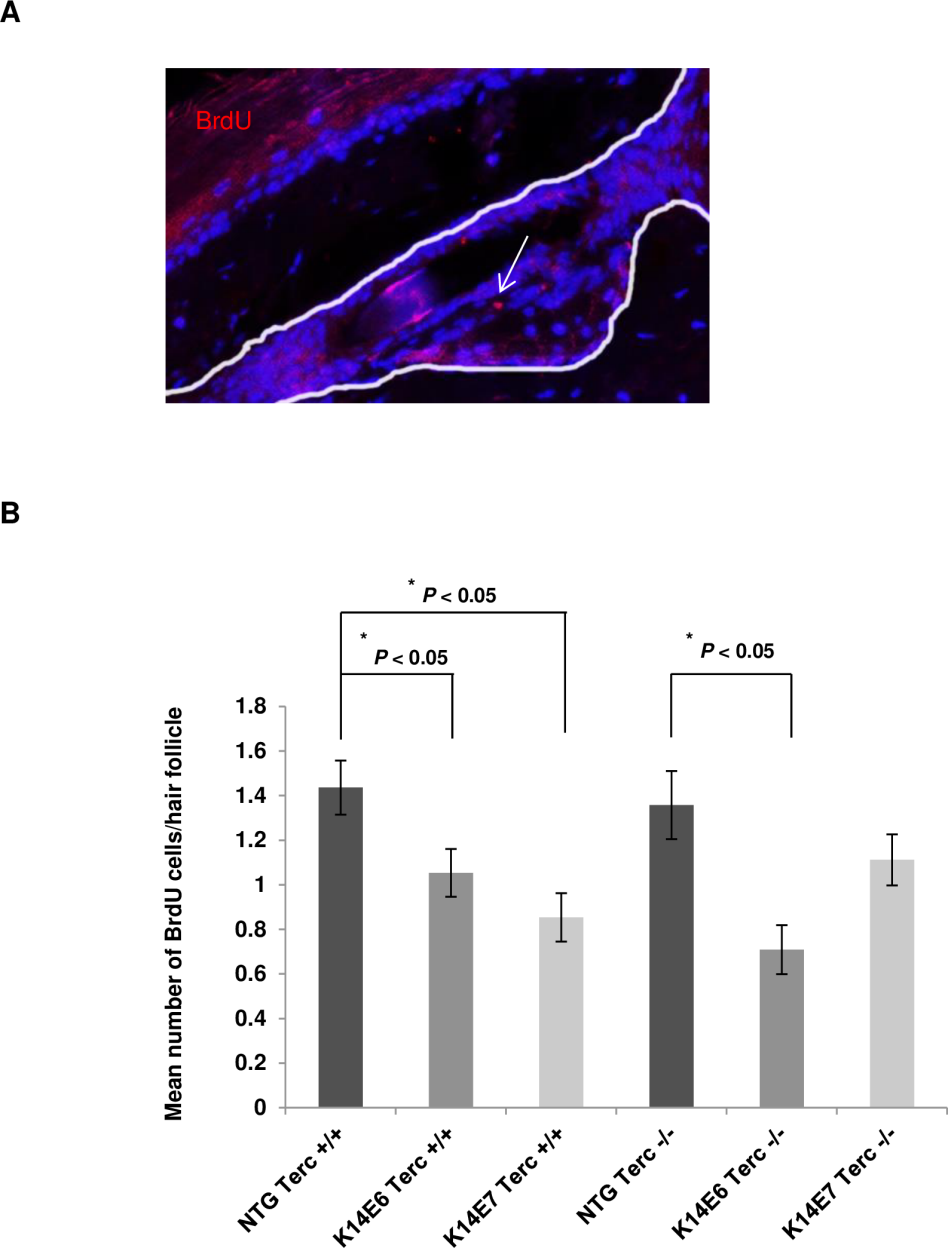
Terc is dispensable for E6 but not E7-mediated effects on hair follicle label- retaining cells. (A) Representative immunofluorescent image of a hair follicle showing BrdU positive cells (red). Counterstaining was done with DAPI (blue). The white line indicates the hair follicle. (B) LRCs were labelled using a BrdU pulse administered shortly after birth and chased until second telogen (71 days old). ~80 hair follicles were selected from at least 3 mice of each genotype (n = 3). The mean number of BrdU positive cells per hair follicle bulge was quantified and plotted for each genotype (columns); bars, SD. All statistical comparisons were performed using a two-sided Wilcoxon rank sum test.

Our lab has previously shown that the HPV16 oncogenes can lead to a reduction in the numbers of label-retaining cells (LRCs) in hair follicles under resting conditions (telogen) attributed to increased proliferation [22]. We examined here the potential role of telomerase activity in these E6 and E7-mediated phenotypes. We performed BrdU pulse-chase assays as previously described [26, 28, 29], and the numbers of label-retaining cells (LRCs) at second telogen (resting phase of hair cycle) were compared in all the genotypes (Fig 4). In the presence of Terc, both E6 and E7 mice showed a reduction in the number of LRCs, a result that is consistent with the previous studies. This increase in proliferation leads to an increase in the number of cell divisions, mobilization of the quiescent cells and loss of BrdU staining.

In the absence of Terc however, there is a rescue in the reduced LRC number phenotype of E7 mice but not of E6, which exhibit a statistically significant reduction of LRCs compared to the non-transgenic Terc deficient mice. E6 and E7 mediate their effects on the hair follicle LRCs via separate pathways [22]. We speculate that the pathway(s) perturbed by E7 render LRCs more sensitive to the lack of functional telomerase.

### Terc is dispensable for HPV-mediated expansion of stemness related markers

**Fig 5:**
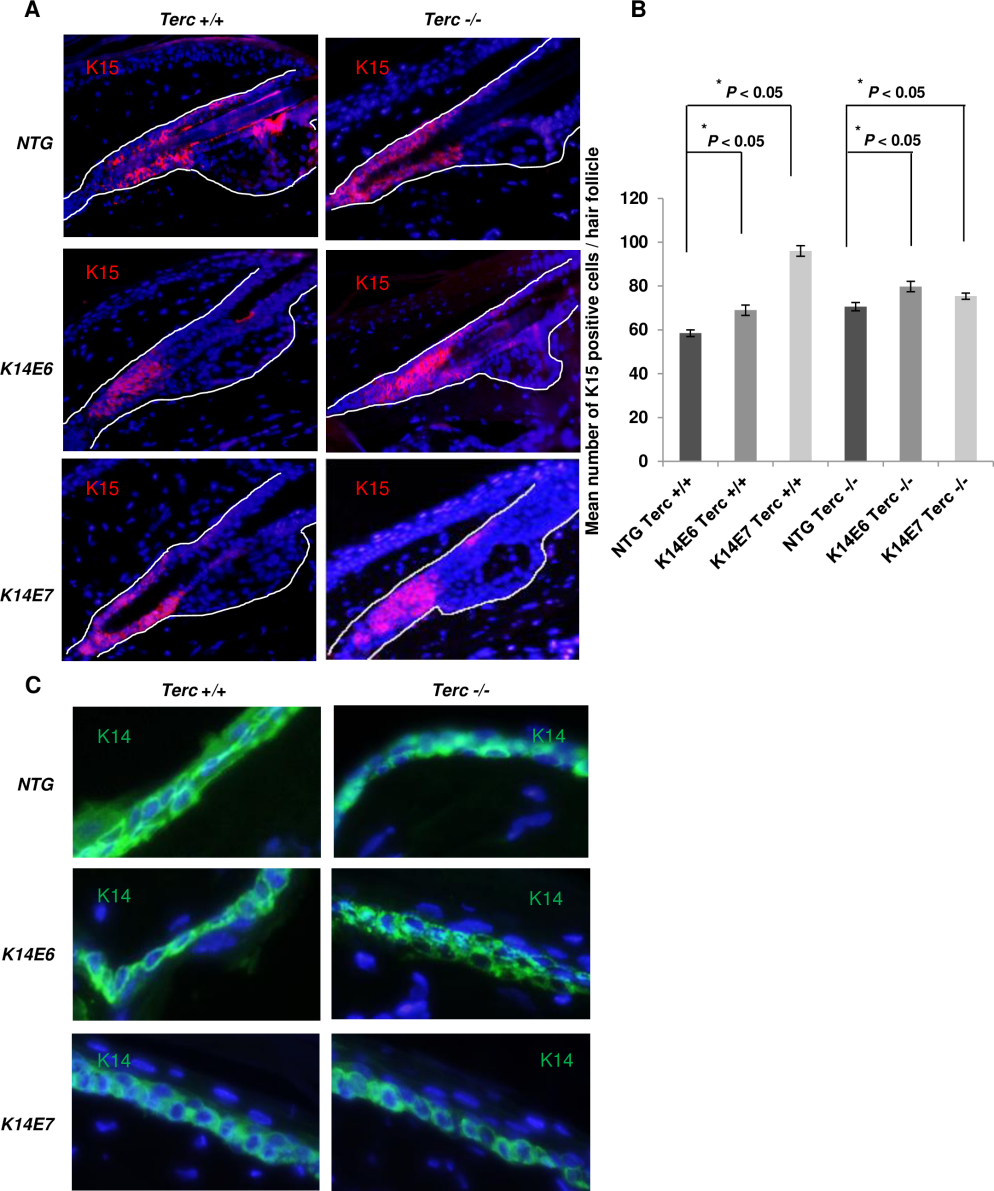
HPV16 oncogene expression causes an expansion of the bulge stemness marker K15. (A) Representative immunofluorescent images of the hair follicles showing K15 staining (red) using a K-15 specific antibody. Counterstaining was done with DAPI (blue). (B) ~80 hair follicles were selected from at least 3 mice of each genotype (n = 3). Mice were 71 days old. The mean number of K15 positive cells of each hair follicle was quantified and plotted for each genotype (columns); bars, SD. All statistical comparisons were performed using a two-sided Wilcoxon rank sum test. (C) Representative immunofluorescent images from the tail epithelium showing K14 expression which correlates with the expression of the E6 and E7 viral oncogenes.

Concurrent with aberrant stem cell mobilization, we have previously reported an expansion of keratin 15 (K15), an endogenous marker of bulge stem cells. Our results showed that the expression of K15 was expanded in both E6 and E7 expressing mice in regions not known to contain stemness markers. This significant increase and expansion was also seen in E6 and E7 expressing mice in a Terc deficient background when compared with the non-transgenic mice of the same background (Fig 5A and 5B). These results indicate that the reduction of LRCs in mice expressing E6 or E7 is due to an increase in the proliferation of the epithelium and an aberrant mobilization and expansion of stem cells even in the absence of the Terc component. Our results also show that the pattern of other differentiation markers such as K14 expression is consistent in all the genotypes obtained and irrespective of the mice mixed background (Fig 5C).

This suggests that the telomere elongation activity of telomerase in mice with sufficiently long telomeres, is not necessary for the mobilization and expansion of stem cells in the epithelium. The role of Tert in this process still needs to be examined in order to evaluate the necessity of the non-telomeric functions of telomerase in those processes.

## Discussion

The high-risk HPVs have been extensively reported to modulate telomere homeostasis, most notably via impinging on the transcription and translation of the telomerase enzyme [13–17]. It has been proposed that the level of regulation seen, particularly by the E6 oncoprotein may point to a way in which E6 alters the “stemness” of infected cells with potential effect on the viral lifecycle and carcinogenesis [13]. To our knowledge, ours is the only study which attempts to decipher the consequences stemming from the interplay of telomere homeostasis and the HPV16 oncogenes *in vivo*.

Our results indicate that telomerase activity is indeed increased when HPV16 E6 is expressed in stratified epithelia. This is not seen when HPV16 E7 is expressed. These findings are consistent with results seen in human keratinocytes transduced with E6 or E7 expressing retroviruses. In human cells the increase in telomerase activity has previously been shown to be mediated via transcriptional and translational pathways: binding to the Tert promoter or by direct binding to the Tert protein [13–17] leading to an increased telomere elongation activity.

Contrary to what is observed in those systems, the expression of E6 and E7 in our transgenic mice does not lead to an elongation of telomeres, as quantified by a sensitive telomere-FISH assay. A likely factor for the discrepancy between previous results and those observed here may be the different levels of oncogene expression. In our system there is a lower expression of E6 and E7, as opposed to the retroviral systems, which is in line with those encountered during human infection [30] and correlates the *in vivo* physiological effects of the oncogenes. While mouse telomeres are longer than those of humans, telomerase preferentially elongates short telomeres and the threshold for what constitutes a short/unprotected telomere also varies accordingly between mouse and human. In physiological contexts (mouse or human), when telomeres are sufficiently long it is not unexpected to see lack of telomere elongation for long periods of time despite elevated levels of telomerase activity [31]. While we cannot exclude the possibility that our results could be attributed to differences between human and mouse telomeres, the mouse has been an incredibly useful model in elucidating the role of telomere homeostasis *in vivo* [24, 32–36].

A number of short-term phenotypes have been previously attributed to the expression of the HPV16 oncogenes *in vivo*, including increased proliferation and aberrant stem cell homeostasis [22, 37]. To determine the contribution of a functional telomerase enzyme in such short-term phenotypes we crossed animals transgenic for the HPV16 oncogenes to animals in which Terc, the RNA component of telomerase, in not expressed. This has previously been shown to reliably ablate the assembly of a functional telomerase enzyme and lack of telomere elongation activity is corroborated by our results. Our system therefore, enables the first study addressing the role of telomerase and HPV16 oncogene interaction in the short-term *in vivo* phenotypes, which may correlate with important aspects of the viral lifecyle [38] and carcinogenesis, where the viral oncogene expression is still relatively low.

We determined that the presence of a functional telomerase enzyme was dispensable for most short-term phenotypes examined. It is conceivable that if elevated telomerase activity (as confirmed in the presence of E6 oncogene expression) has a role in the viral lifecycle, it may relate to aspects of viral replication or maintenance which are not directly probed in our system. The same would be true, in the case of effects which require the synergistic action of the two oncogenes [39]. Potential roles in carcinogenesis may exist in later steps during that process.

Surprisingly, the ability of E7 to contribute to LRC reduction was found to be in part dependent on the presence of Terc and functional telomerase assembly. Since stem cells are often characterized by increased telomerase activity we propose that in a context where there is increased stem cell mobilization (as has been previously shown for E7) the inability to elongate telomeres may be of increased importance. Increased mobilization of stem cells may “sensitize” them to a lack of functional telomerase.

There also exists controversial evidence for non-canonical functions of components of the telomerase enzyme, in particular Tert [40]. Since such non-canonical effects of the enzyme have been heavily debated we chose to focus our studies on the canonical effects, the main and evolutionarily conserved function of the enzyme. Thus, we performed our studies using a mouse in which Terc has been eliminated. Extratelomeric effects have not been reported for terc in mammalian systems or in tissue types relevant to the HPV replication [41]. Further studies would need to be performed to examine potential non-canonical role of Tert expression in the context of HPV oncogene expression *in vivo*.
